# Data on copper level in the blood of patients with normal and abnormal angiography

**DOI:** 10.1016/j.dib.2016.08.021

**Published:** 2016-08-18

**Authors:** Leila Amiri, Ali Movahed, Dariush Iranpour, Afshin Ostovar, Alireza Raeisi, Mozhgan Keshtkar, Najmeh Hajian, Sina Dobaradaran

**Affiliations:** a Department of Environmental Health Engineering, Faculty of Health, Bushehr University of Medical Sciences, Bushehr, Iran; b Bushehr University of Medical Sciences, The Persian Gulf Tropical Medicine Research Center, Biochemistry Group, Bushehr, Iran; c Bushehr University of Medical Sciences, Department of Cardiology, Faculty of Medicine, Bushehr, Iran; d The Persian Gulf Tropical Medicine Research Center, The Persian Gulf Biomedical Sciences Research Institute, Bushehr University of Medical Sciences, Bushehr, Iran; e The Persian Gulf Marine Biotechnology Research Center, The Persian Gulf Biomedical Sciences Research Institute, Bushehr University of Medical Sciences, Bushehr, Iran; f Systems Environmental Health, Oil, Gas and Energy Research Center, The Persian Gulf Biomedical Sciences Research Institute, Bushehr University of Medical Sciences, Bushehr, Iran

**Keywords:** Angiography, Cardiovascular disease, Copper, Bushehr

## Abstract

In this data article, we measured the levels of copper in the blood of patients undergoing coronary angiography. The samples were taken from patients with cardiovascular disease in Bushehr׳s university hospital, Iran. Patients were divided in two groups: normal angiography and abnormal angiography. After the chemical digestion of samples, the concentration levels of Cu in both groups were determined by using inductively coupled plasma optical spectrometry (ICP-OES).

**Specifications Table**

**Table:** 

*Subject area*	*Medicine*
*More specific subject area*	*Heart, biochemistry heart*
*Type of data*	*Table*
*How data was acquired*	*ICP-OES (SPECTRO (Germany), Spectro arcos)*
*Data format*	*Preliminary data, analyzed*
*Experimental factors*	*The samples were from patients with cardiovascular disease, with normal and abnormal angiography. After sampling, the blood serum was isolated by centrifugation. Then they were stored at −80°C until final analysis*
*Experimental features*	*Determine the level of copper concentration level in blood samples of patients with cardiovascular disease*
*Data source location*	*Bushehr, Iran*
*Data accessibility*	*Data is with this article.*

**Value of the data**

• The data can be used in other studies to determine trace elements in the blood.
• The data can be used to evaluate the effect of trace elements on heart function.
• The data can be used for further studies on the trace element effects on other human organs as well as the possible role of trace elements in preventing diseases.

## 1 Data

We measured concentration levels of Cu in blood samples from patients with cardiovascular disease. General data on patients involved in present study is shown in Table 1. The concentration levels of Cu in patients with normal angiography were in the range of 24.01–135.76 µg/dL with a mean concentration level of 54.14 µg/dL. The concentration levels of Cu in patients with abnormal angiography were in the range of 33.6–221.3 µg/dL with a mean concentration level of 60.9 µg/dL (Table 2). Chi- Square analysis also showed that between all characteristics shown in Table 1, only age had statistically significant relationship with angiography type (*p*-value=0.033).

## 2 Experimental design, materials and methods

Blood samples were taken from a total of 120 patients, including the patients with normal angiography (*N*=60) and patients with abnormal angiography (*N*=60). These individuals were randomly selected from cardiovascular patients undergoing coronary angiography, at university heart hospital of Bushehr. ECG, echo and angiography were performed for both groups. These who had normal ECG, echo and normal angiography, were placed in the control group, and so people with ECG, echo and abnormal angiogram were classified in the category of abnormal. After sampling, the blood serum was isolated by centrifuge. Then they were stored at −80 °C until final analysis. For digestion of samples, 2.5 ml of serum sample was mixed with 2 ml of HNO_3_ and 1 ml of H_2_O_2_. The resulting mixture was placed in an oven for 2 h at 50 °C. After that, 1 ml of concentrated nitric acid and a few drops of H_2_O_2_ were added to the solution. Then solution was placed on a hot plate, until clear solution was obtained. The resulting clear solution was cooled down at room temperature and 5 ml HNO_3_ 0.1 N, was added and a volume of 50 ml from sample was prepare by using deionized water [1]. After chemical digestion and preparation, the samples were read by using ICP-OES [2], [3], [4], [5].

## Figures and Tables

**Table 1:** General characteristics of patients with both normal angiography and abnormal angiography.

**General characteristics of patients**		**Angiography**	
		**Normal**	**Abnormal**
**Gender**	Male	50.7%	49.3%
	Female	49.0%	51.0%
	<40 year	87.5%	12.5%
	41–54 year	56.0%	44.0%
**Age group**	55–64 year	50.9%	49.1%
	65–74 year	28.6%	71.4%
	>75 year	20.0%	80.0%
**BMI**	Normal	45.2%	54.8%
	Overweight	52.5%	47.5%
	Obese	50.0%	50.0%
**Mitral regurgitation**	Positive	43.7%	56.3%
	Negative	63.9%	36.1
**Family history of heart disease**	Yes	48.1%	51.9%
	No	53.8%	46.2%
**Duration of having heart disease**	*x*<1 year	51.8%	48.2%
	1–5 year	50.0%	50.0%
	5–10 year	0.0%	100.0%
**Hypertension**	No	35.9%	64.1%
	Yes	57.7%	42.3%
**Diabetes**	Yes	38.5%	61.5%
	No	52.7%	47.3%
**Smoking**	Yes	59.2%	40.8%
	No	44.1%	55.9%

**Table 2:** Mean, maximum and minimum concentration level (µg/dL) of Cu in patient with normal angiography and abnormal angiography.

**Cu level in blood samples**	**Normal angiography**	**Abnormal angiography**
**Number**	60	60
**Minimum**	24.01	33.6
**Maximum**	135.76	221.3
**Mean**	54.14	60.9
**Sig.**	0.096	0.096
